# Bioabsorbable implants are a viable alternative to traditional metallic implants in orthopaedic surgery: a systematic review and meta-analysis

**DOI:** 10.1016/j.jor.2025.06.005

**Published:** 2025-06-14

**Authors:** Benjamin Blackman, Stefan Okunbor, Aubrie M. Sowa, Jake M. McDonnell, Tayler D. Ross, Brian Rigney, Stacey Darwish, Joseph S. Butler

**Affiliations:** a School of Medicine, University of Limerick, Limerick, Ireland; b National Spinal Injuries Unit, Mater Misericordiae University Hospital, Dublin, Ireland; c School of Medicine, University College Cork, Cork, Ireland; d School of Medicine, University College Dublin, Dublin, Ireland; e Trinity Centre for Biomedical Engineering, Trinity Biomedical Sciences Institute, Trinity College Dublin, The University of Dublin, Dublin, Ireland; f Division of Orthopaedic Surgery, Department of Surgery, University of Toronto, Toronto, Ontario, Canada; g Department of Orthopaedics, St. Vincent's University Hospital, Dublin, Ireland

**Keywords:** Bioabsorbable, Biodegradable, Implants, Orthopaedic, Metal

## Abstract

**Introduction:**

This systematic review and meta-analysis of randomized controlled trials (RCTs) aims to compare clinical outcomes and complication profiles of bioabsorbable versus metallic implants in orthopaedic surgery.

**Methods:**

Four databases (PubMed, MEDLINE, EMBASE, CINAHL) were searched from inception to April 8, 2025, for RCTs comparing bioabsorbable and metal implants in orthopaedic procedures. Data were extracted on demographics, implant type, complications, and outcomes. Meta-analyses using random-effects models were performed for pooled comparisons, and heterogeneity was quantified using the *I*^*2*^ statistic. Risk ratios (RR) and mean differences (MD) were calculated with 95 % confidence intervals (CI).

**Results:**

Twenty-seven RCTs involving 1437 patients (738 bioabsorbable, 699 metallic) were included. Overall complication rates were similar between groups (RR: 1.05, 95 % CI: 0.62–1.77, *I*^*2*^ = 49.8 %, p = 0.85). Bioabsorbable implants had a 15.5 % complication rate compared to 13.3 % for metallic implants. Newer materials showed lower complication rates of 2.5 % for PLLA-HA and 5 % for magnesium compared to 16.5 % for other compounds. There were no foreign body reactions in the studies using newer materials. There was a statistically significant decrease in surgical site infections in the bioabsorbable group (RR: 0.36, 95 % CI: 0.17, 0.79, *I*^*2*^ = 0 %, p = 0.02). No significant differences were found regarding hardware failure, pain and functional deficiency, and healing rates. Subgroup analysis of ankle procedures (n = 5) showed a higher complication rate in the bioabsorbable group (RR: 3.75, 95 % CI: 1.02, 13.8, *I*^*2*^ = 25 %, p = 0.05), influenced by one study reporting a 33.7 % foreign body reaction rate. Anterior cruciate ligament reconstruction, foot, and upper limb subgroups showed comparable outcomes.

**Conclusion:**

Bioabsorbable implants provide comparable complication rates and clinical outcomes to metallic implants across orthopaedic subspecialties. Outcomes may vary by anatomical site and implant generation, with newer bioabsorbable materials demonstrating promising safety profiles. Further long-term studies are warranted to evaluate their performance in high-stress applications and guide clinical decision-making.

**Level of evidence:**

Level I – Systematic Review of Randomized Controlled Trials.

## Highlights

• Complication rates were similar between groups, with newer materials such as PLLA-HA and Mg leading to fewer complications.
• There were significant reductions in surgical site infections in the bioabsorbable group.
• Bioabsorbable implants provided comparable outcomes, although results varied by anatomical site and material generation.

## Abbreviations:

**ACL**: Anterior Cruciate Ligament
**AOFAS**: American Orthopaedic Foot and Ankle Society
**DASH**: Disabilities of the Arm, Shoulder, and Hand
**FBR**: Foreign body reaction
**NiTi**: Nickel-Titanium
**PGA**: Polyglycolic Acid
**PLLA**: Poly-L-Lactic Acid
**PLLA-HA**: Poly-L-lactic acid + hydroxyapatite
**PRISMA**: Preferred Reporting Items for Systematic Reviews and Meta-Analyses
**PROM**: Patient-Reported Outcome Measure
**RCT**: Randomized Controlled Trial
**RR**: Risk Ratio
**SS**: Stainless Steel
**SSI**: Surgical Site Infection
**Ti**: Titanium
**VAS**: Visual Analog Scale

## 1 Introduction

The use of metal implants in surgery dates back to the 16th century,^1^ with metallic biomaterials assuming a vital role in orthopaedic surgery. Over the years, advances in implant surgery have been made with the integration of various metals and alloys, including non-degradable stainless steel (SS), titanium (Ti)-based alloys, cobalt (Co)-based alloys, and nickel-titanium (NiTi) alloys.^2^ These metallic materials have many properties that make them suitable for orthopaedic procedures, such as high strength, fracture toughness, hardness, corrosion resistance, and biocompatibility.^3^

During the 1950s, surgical implants evolved to include non-metal materials, including biodegradable and non-biodegradable ceramics and polymers.^1^ First-generation bioabsorbable implants were composed of polyglycolic acid (PGA). The swift degradation of this product resulted in reports of inflammatory foreign-body reactions (FBR). This phenomenon drove the production of numerous devices utilizing poly-L-lactic acid (PLLA), which is characterized by an extended degradation time, or a combination of PGA and PLLA. Additionally, bioabsorbable metals such as magnesium, iron, and zinc can be used, as these are stronger than bioabsorbable polymers.^4^, ^5^ Bioabsorbable devices offer several advantages compared to traditional non-absorbable metallic devices as they minimize the necessity for surgical removal. In contrast to metallic implants, bioabsorbable implants create minimal artifacts in imaging studies, thereby enhancing the postoperative evaluation of fragment healing.^6^

Metallic implants have established a high benchmark in orthopaedics due to their durable mechanical qualities and reliable performance within the body. Nonetheless, their enduring nature may require surgical interventions for implant removal, especially in younger or more active individuals. Bioabsorbable implants present a viable alternative; nevertheless, they must achieve the mechanical dependability and predictability of modern metal implants.

Although evidence evaluating these implants exists, there remains a gap in thorough research that synthesize these diverse clinical results. This review aims to compare the clinical outcomes and complication profiles of bioabsorbable and metallic implants to determine if bioabsorbable implants are a viable alternative in orthopaedic surgery.

## 2 Methods

### 2.1 Search strategy and study selection

Two independent reviewers conducted a systematic literature search following the Preferred Reporting Items for Systematic Reviews and Meta-Analyses (PRISMA) guidelines.^7^ A thorough search was performed in the Embase, PubMed, Medline, and CINAHL databases for studies published until April 8, 2025. The search strategy utilized combinations of medical subject headings (MeSH) and relevant keywords concerning implants and outcomes, specifically: ("Absorbable Implants" OR "bioabsorbable" OR "biodegradable") AND ("Orthopaedic Procedures" OR "orthopaedics" OR "orthopaedics" OR "orthopaedic surgery" OR "orthopaedic surgery") AND ("Fracture Fixation, Internal" OR "traditional implants" OR "non-absorbable implants" OR "metal implants" OR "titanium") AND ("Randomized Controlled Trial" OR "Comparative Study"). The reference lists of retrieved full-text articles were reviewed and screened to identify additional studies that met inclusion criteria.

### 2.2 Screening

Title and abstract screening were conducted independently by two authors, with conflicts resolved through consultation with a senior author. During the full‐text screening stage, studies were independently reviewed by the same two authors, and disagreements were resolved in the same manner.

### 2.3 Quality assessment

Quality assessment of randomized controlled trials (RCTs) was conducted using the Cochrane Risk of Bias 2 (RoB-2) tool. Five domains are evaluated^1^: bias from the randomization process,^2^ bias due to deviations from intended interventions,^3^ bias due to missing outcome data,^4^ bias in measurement of the outcome, and^5^ bias in selection of the reported result. Each domain is categorized as either^1^ low risk of bias,^2^ some concerns, or^3^ high risk of bias. Overall risk of bias was considered to be ‘low risk’ for studies assessed to be at low risk of bias in all domains, ‘some concerns’ if the study raised concerns in at least one domain, and ‘high risk’ if the study was judged to be high risk of bias in at least one domain, or if the study raised some concerns in multiple domains.^8^

### 2.4 Eligibility criteria

The inclusion criteria consisted of the following: (i) orthopaedic procedure, (ii) randomized controlled trials, (iii) involvement of patients with bioabsorbable implants, (iv) involvement of patients with metal implants, and (v) reporting of human outcomes/results. The exclusion criteria comprised (i) non-RCTs, including case reports and case series, (ii) non-human studies, such as animal studies and in vitro research, (iii) studies lacking clear outcome reporting, and (iv) non-English studies without accessible translations**.**

### 2.5 Data extraction

The data extraction process was systematic, uniform, and performed independently by two reviewers. Data on sample sizes, types of orthopaedic implants, complications, and clinical outcomes were collected. Microsoft Excel was employed to organize and classify this data for consistent compilation and analysis. The meticulous extraction approach allowed for thorough evaluating of study quality, risk of bias, and statistical analysis of data in RStudio.

### 2.6 Outcomes analyzed and statistics

Clinical outcomes, including surgical site infections (SSI), hardware failure, pain or functional impairments, and indicators of healing, were systematically evaluated, in addition to patient-reported outcomes measures (PROM). The examined parameters include: persistent pain and Disabilities of the Arm, Shoulder, and Hand (DASH) scores for upper limbs; time to union and operative durations for ankle operations; postoperative pain and the American Orthopaedic Foot and Ankle Society (AOFAS) scores for foot surgeries; Lysholm scores and Lachman test outcomes for Anterior Cruciate Ligament (ACL) surgeries. Statistical analyses were conducted using RStudio (version 2024.12.0), an integrated development environment for R. Variability among studies was addressed using a random-effects model, and heterogeneity was quantified with the *I*^*2*^ statistic. Forest plots were generated to visually represent effect sizes and confidence intervals, enabling a direct comparison between the groups across all studies included**.**

## 3 Results

### 3.1 Literature search

**Fig. 1:**
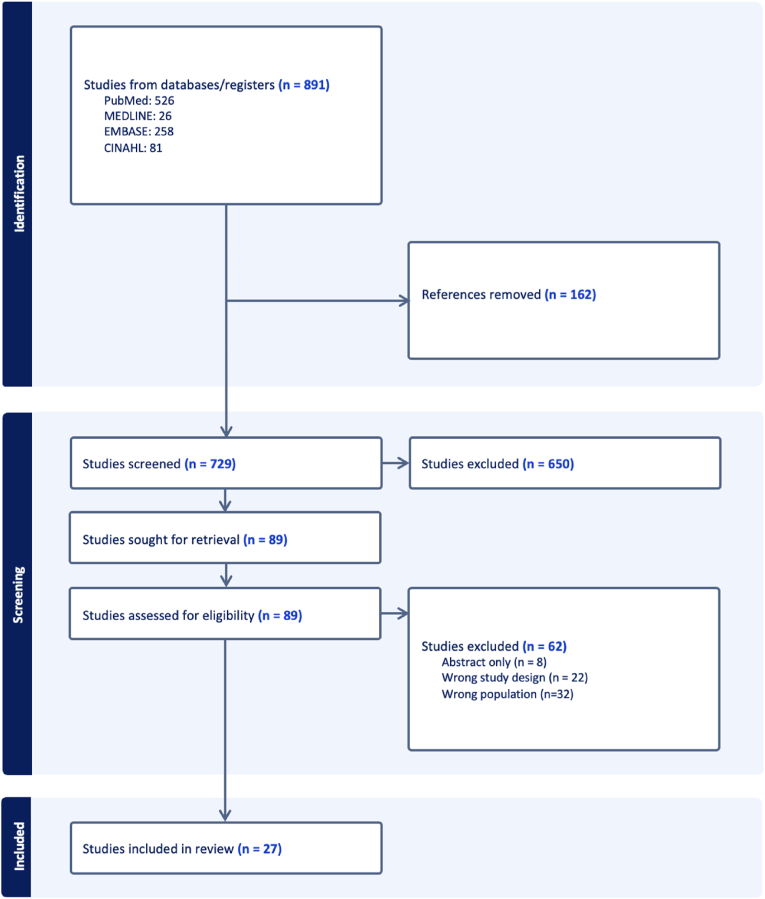
Preferred Reporting Items for Systematic Reviews and Meta-analyses (PRISMA) flow diagram representing a systematic review on bioabsorbable versus conventional implants in orthopaedic surgery.

The initial literature search yielded 891 studies, of which 162 were duplicates and were removed. Of the remaining 729 articles, 650 were removed following title and abstract screening. Systematic screening and eligibility assessment yielded 27 full‐text studies that satisfied inclusion criteria (Fig. 1).

### 3.2 Study quality

All studies included in this systematic review were Level 1 evidence, as only RCTs were

**Fig. 2:**
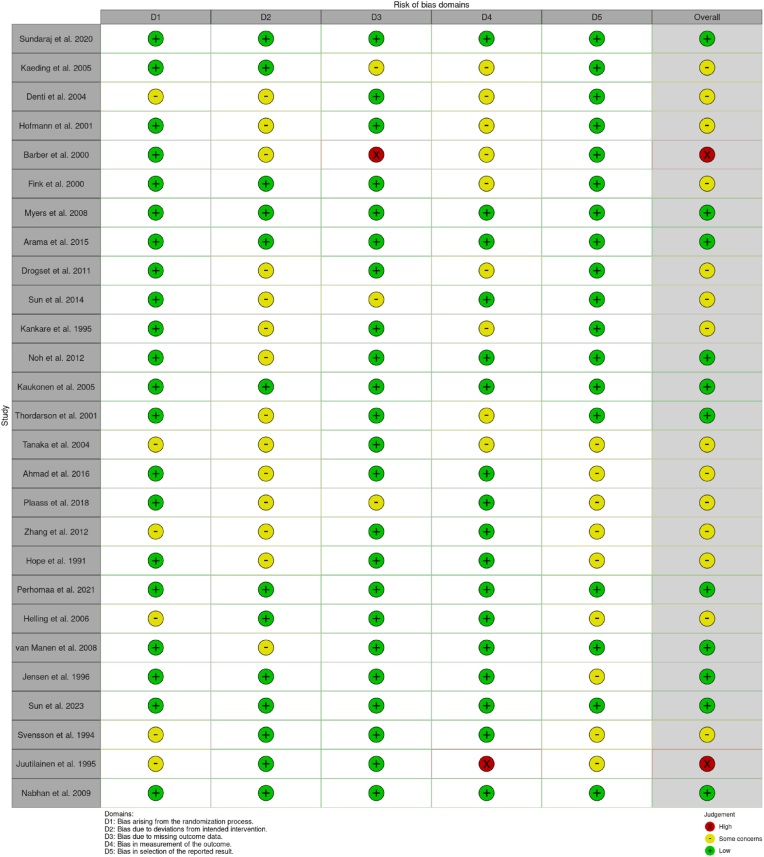
Traffic light plot demonstrating the risk of bias domains for the twenty-seven randomized controlled trials.

Included. The Cochrane RoB-2 tool identified^11^ low risk of bias studies, 2 high risk of bias studies, and some concerns about bias in 14 studies. The risk of bias for individual domains is shown in Fig. 2.

### 3.3 Study characteristics

This review included 1437 patients, of whom 738 were treated with bioabsorbable implants and 699 with traditional metal implants. Studies assessing bioabsorbable versus metallic implants in ACL surgery^9^, ^10^, ^11^, ^12^, ^13^, ^14^, ^15^, ^16^, ^17^ were the most prevalent, making up nine studies. Five studies examined the ankle.^18^, ^19^, ^20^, ^21^, ^22^ Four studies examined the foot,^23^, ^24^, ^25^, ^26^ three the forearm,^27^, ^28^, ^29^ and one the elbow.^30^ One study each examined the wrist,^31^ hand,^32^ hip^33^ and spine.^34^ One study looked at multiple fracture sites.^35^

### 3.4 Complications

**Fig. 3:**
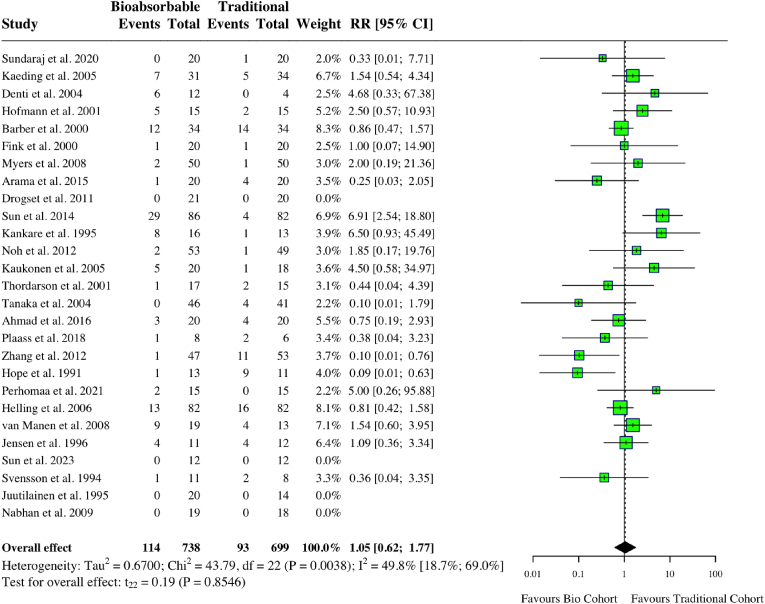
Forest plot (random effects) with accompanying risk ratio calculations and 95 % confidence intervals for total complications comparing bioabsorbable and traditional implants.

Various complications were reported in the studies, including, SSIs, hardware failure, recurrent pain and functional deficiency, and impaired healing (i.e. malunion, nonunion). A meta-analysis of 27 studies comparing bioabsorbable and traditional implants found no statistically significant difference in total complications (RR: 1.05, 95 % CI: 0.62, 1.77, *I*^*2*^ = 49.8 %, p = 0.85) (Fig. 3). The total complication rate for bioabsorbable implants was 15.5 %, compared to 13.3 % for metallic implants. The overall complication rate in Poly-L-lactic acid and hydroxyapatite (PLLA-HA) compounds was 2.5 %.^10^^,^^17^ The overall complication rate for magnesium implants was 5 %.^24^^,^^33^ The overall complication rate for all other bioabsorbable compounds was 16.5 %.

**Fig. 4:**
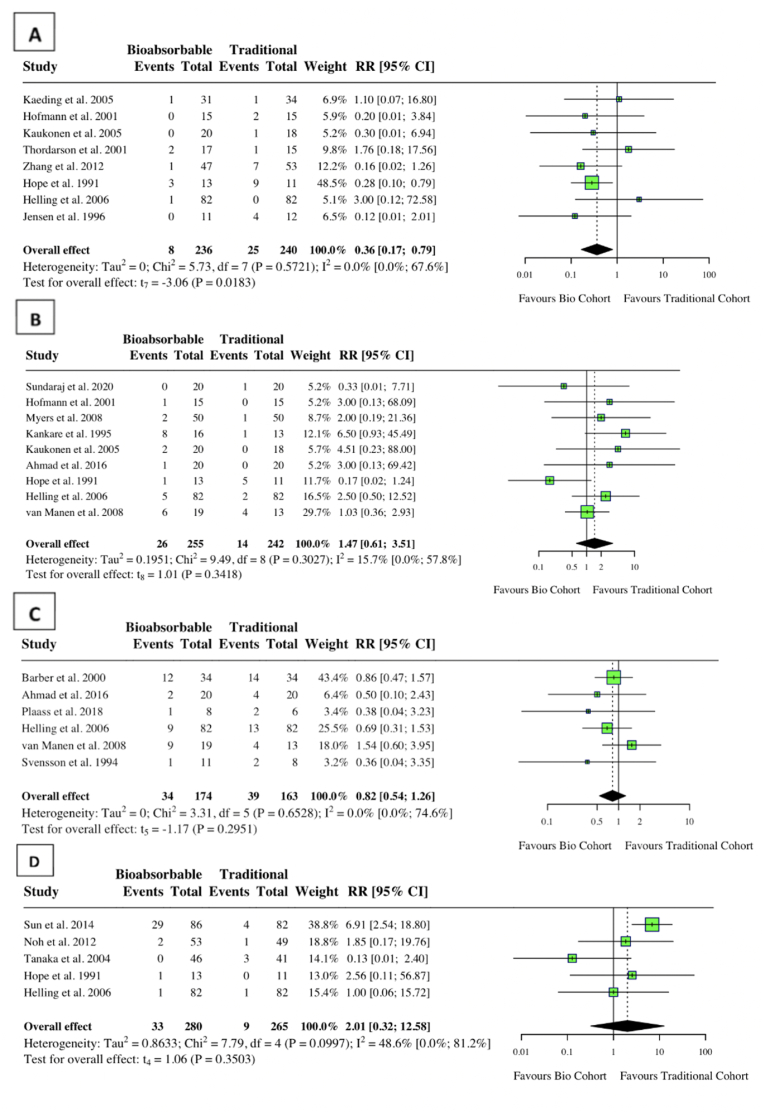
Forest plot (random effects) with accompanying risk ratio calculations and 95 % confidence intervals for (A) Surgical Site Infections, (B) Hardware Failure, (C) Pain and Functional Deficiency, (D) Healing.

There was a statistically significant decrease in surgical site infections in the bioabsorbable group (RR: 0.36, 95 % CI: 0.17, 0.79, *I*^*2*^ = 0 %, p = 0.02) (Fig. 4). Neither group had a statistical difference in hardware failure, pain and functional deficiency, and healing rates (Fig. 4).

### 3.5 Subgroup analysis

#### 3.5.1 Ankle

**Fig. 5:**
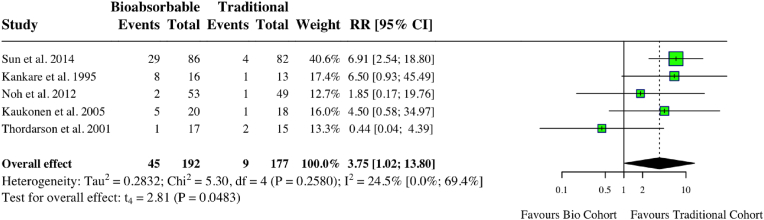
Forest plot (random effects) with accompanying risk ratio calculations and 95 % confidence intervals for complications comparing bioabsorbable and traditional implants in ankle studies.

A meta-analysis of five studies assessing complications in ankle cohorts found a statistically significant increase in complications for those undergoing bioabsorbable fixation (RR: 3.75, 95 % CI: 1.02, 13.8, *I*^*2*^ = 25 %, p = 0.05) (Fig. 5). One study found that 33.7 % of those undergoing bioabsorbable fixation had FBRs 21. When this study was excluded, the difference was no longer statistically significant (RR: 2.48, 95 % CI: 0.38, 16.3, *I*^*2*^ = 14 %, p = 0.22). A meta-analysis of two studies investigating time to union found no difference between groups (MD: 0.30, 95 % CI: 3.98, 4.59, *I*^*2*^ = 83 %, p = 0.53). Operative times among these two studies did not differ (MD: 1.27, 95 % CI: 23.3, 25.85, *I*^*2*^ = 99 %, p = 0.63). Another study found a statistically significant decrease in operating time in the metallic group; however, the bioabsorbable group had a higher return to the sport of 85 % compared to 66.7 % in the metallic group 19. Another study found no statistically significant difference in AOFAS scores between groups at 12 months and had a 4.1 % non-union rate in the bioabsorbable group compared to 0 % in the metallic group 20. Individual study characteristics and outcomes can be seen in Table 1.

**Table 1:** Study characteristics and outcomes for ankle studies.

Author (Year)	Patients Metal: M Bio: B	Mean follow-up time (months) Metal: M Bio: B	Lost to follow-up (%) Metal: M Bio: B	Mean age (years) Metal: M Bio: B	Implant material Metal: M Bio: B	Complications (%) Metal: M Bio: B	Operation Time (mins) Metal: M Bio: B
Sun (2014)	M: 82 B: 86	M: 55.9 B: 55.7	M: 10 B: 6	M: 37.1 B: 39.7	M: Titanium B: PLLA	M: 4 B: 29	M: 83.7 B: 84.5
Thordarson (2001)	M:15 B: 17	M: 10.5 B: 11.4	M: 4 B: 4	M: 24.2 B: 34.7	M: Stainless steel B: PLA	M: 2 B: 1	NR
Kaukonen (2005)	M: 18 B: 20	M: 35 B: 35	M: 2 B: 0	M: 45.6 B: 43.1	M: NR B: PLLA	M: 1 B: 3	M: 64 B: 79
Kankare (1995)	M: 12 B: 16	M: 25.2 B: 35.7	M: 6 B: 11	M: 46 B: 46	M: NR B: PGA	M: 1 B: 8	M: 53 B: 48
Noh (2012)	M: 53 B: 49	M: 19.7 B: 19.7	M: 3 B: 4	NR	M: NR B: PLLA	M: 1 B: 2	M: 30.2 B: 56.4

PLLA: Poly-L-lactic acid.

PGA: Polyglycolic acid.

NR: Not reported.

#### 3.5.2 ACL

**Fig. 6:**
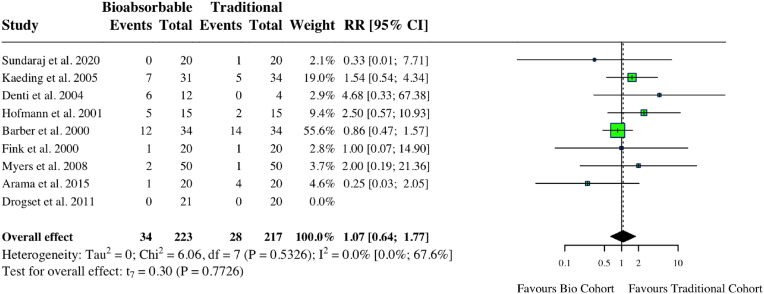
Forest plot (random effects) with accompanying risk ratio calculations and 95 % confidence intervals for complications comparing bioabsorbable and traditional implants in anterior cruciate ligament studies.

**Fig. 7:**
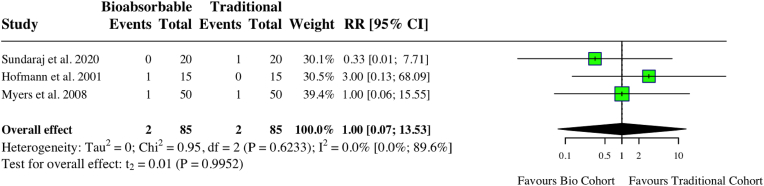
Forest plot (random effects) with accompanying risk ratio calculations and 95 % confidence intervals for graft failure comparing bioabsorbable and traditional implants in anterior cruciate ligament studies.

**Fig. 8:**
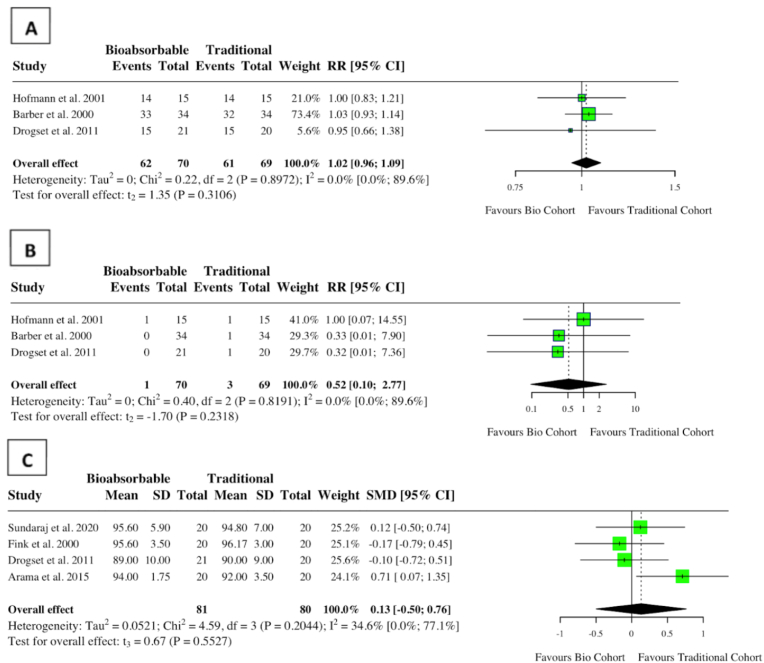
Forest plot (random effects) with accompanying risk ratio calculations/mean differences and 95 % confidence intervals for (A) Lachman ≤1, (B) Lachman ≥2, (C) Lysholm score.

A meta-analysis of nine studies assessing complications in ACL cohorts found no increase in complications for those undergoing bioabsorbable fixation (RR: 1.07, 95 % CI: 0.64, 1.77, *I*^*2*^ = 0 %, p = 0.77) (Fig. 6). There was no difference in graft failure among the two groups (RR: 1.00, 95 % CI: 0.07, 13.53, *I*^*2*^ = 0 %, p = 0.99) (Fig. 7). A meta-analysis of three studies examining the prevalence of a negative Lachman (≤ Grade 1) post-ACL reconstruction found no difference regardless of implant type (RR: 1.02, 95 % CI: 0.96, 1.09, *I*^*2*^ = 0 %, p = 0.31). There was also no difference in Lachman ≥ grade 2 (RR: 0.52, 95 % CI: 0.10, 2.77, *I*^*2*^ = 0 %, p = 0.23). Lysholm scores were similar among both groups (MD: 0.13, 95 % CI: 0.50, 0.76, *I*^*2*^ = 35 %%, p = 0.55) (Fig. 8). One study found a statistically significant improvement in post-operative hyperextension limit in the titanium group, of 3.8 (2.7), compared to 2.1 (2.4) in the bioabsorbable group.^15^ One study reported a higher failure rate in the PLLA group (5.9 % compared to 4 %).^16^ Individual study characteristics and outcomes can be seen in Table 2.

**Table 2:** Study characteristics and outcomes for anterior cruciate ligament studies.

Author (Year)	Number of Patients Metal: M Bio: B	Mean follow-up time (months)	Lost to follow-up	Mean age (years) Metal: M Bio: B	Graft Type	Implant material Metal: M Bio: B	Complications (%) Metal: M Bio: B	Lysholm Score (SD) Metal: M Bio: B
Sundaraj (2020)	M: 20 B: 20	156	6	M: 29 B: 33	4-strand autologous gracilis and semitendinosus graft	M: Titanium B: PLLA-HA	M: 1 B: 0	M: 94.8^7^ B: 95.6 (5.9)
Kaeding (2005)	M: 34 B: 31	24	32	M: 26.9 B: 26.9	BPTB autograft	M: Titanium B: PLLA	M: 5 B: 7	NR
Denti (2004)	M: 4 B: 16	24	0	M: 28.6 B: 28.6	BPTB autograft	M: Titanium B: PLLA, PGA, PLA	M: 0 B: 6	NR
Hofmann (2001)	M: 15 B: 15	27.95	0	M: 29.9 B: 33.3	BPTB autograft	M: Titanium B: PLA	M: 2 B: 5	NR
Fink (2000)	M: 20 B: 20	11.125	3	M: 29.6 B: 26.8	NR	M: Titanium B: PLLA	M: 1 B: 1	M: 96.2^3^ B: 95.6 (3.5)
Myers (2008)	M: 50 B: 50	12.5	10	M: 30.7 B: 29.6	Hamstring autograft	M: Titanium B: PLLA	M: 1 B: 2	M: 91.7 B: 90.5
Drogset (2011)	M: 20 B: 21	90	5	NR	BPTB autograft	M: Titanium B: PLLA	M: 0 B: 0	M: 90^9^ B: 89^10^
Barber (2000)	M: 34 B: 34	35	46	M: 30 B: 29	Tendon autograft	M: Titanium B: PLLA	M: 14 B: 12	M: 95 B: 94
Arama (2015)	M: 20 B: 20	60	0	M: 33 (7.2) B: 29 (6.5)	4-stranded autologous gracilis and semitendinosus graft	M: Titanium B: PLLA-HA	M: 4 B: 1	M: 92 (3.5) B: 94 (1.75)

PLLA: Poly-L-lactic acid.

PGA: Polyglycolic acid.

PLLA-HA: Poly-L-lactic acid + hydroxyapatite.

PLA: Polylactic acid.

BPTB: Bone-patellar tendon-bone.

NR: Not reported.

#### 3.5.3 Foot

**Fig. 9:**
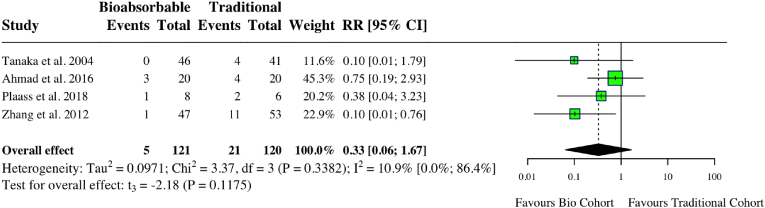
Forest plot (random effects) with accompanying risk ratio calculations and 95 % confidence intervals for complications comparing bioabsorbable and traditional implants in foot studies.

A meta-analysis of four studies assessing complications in foot studies found no difference between groups (RR: 0.33, 95 % CI: 0.06, 1.67, *I*^*2*^ = 11 %, p = 0.12) (Fig. 9). A meta-analysis of three studies examining postoperative pain after foot operations showed no statistically significant difference regardless of implant type (RR: 0.56, 95 % CI: 0.06, 5.26, *I*^*2*^ = 31 %, p = 0.38). A meta-analysis of two studies assessing the AOFAS score in foot operations also showed no difference (MD: 0.09, 95 % CI: 1.30, 1.48, *I*^*2*^ = 0 %, p = 0.57). One study found no statistically significant difference in VAS scores between the steel screws and PLA screws group.^23^ Another found an equal improvement in VAS pain scores in both the magnesium and titanium screw groups.^24^ Individual study characteristics and outcomes can be seen in Table 3.

**Table 3:** Study characteristics and outcomes for foot studies.

Author (Year)	Patient Population	Number patients Metal: M Bio: B	Mean follow-up time (months) Metal: M Bio: B	Lost to follow-up (%)	Female (%) Metal: M Bio: B	Mean age (years) Metal: M Bio: B	Implant type Metal: M Bio: B	Complications (%) Metal: M Bio: B	Post-op Pain (n patients) Metal: M Bio: B	FAAM Metal: M Bio: B	AOFAS Metal: M Bio: B
Tanaka (2004)	Rheumatoid forefoot	M: 29 B: 33	M: 92.4 B: 92.4	3	M: 47 B: 47	M: 55 B: 55	M: Kirschner wire B: PLLA	M: 3 B: 0	M: 12 B: 12	NR	NR
Ahmad (2016)	Lisfranc Injuries	M: 20 B: 20	M: 40.5 B: 36.3	0	M: 11 B: 12	M: 37.1 B: 40.3	M: Stainless Steel B: PLA	M: 4 B: 3	NR	M: 89.6 B: 91.2	NR
Plaass (2018)	Symptomatic hallux valgus, chevron osteotomy	M: 8 B: 6	M: 37.2 B: 38.16	12	M: 6 B: 8	M: 52 B: 56	M: Titanium B: Magnesium	M: 2 B: 1	M: 3 B: 0	M: 95 B: 94	M: 92 B: 95
Zhang (2012)	Calcaneal fracture	M: 53 B: 47	M: 23 B: 23	0	M: 14 B: 12	M: 40 B: 41	M: Metal plate B: PLA screw	M: 10 B: 1	M: 4 B: 1	NR	M: 71.6 B: 72.3

PLLA: Poly-L-lactic acid.

PLA: Polylactic acid.

AOFAS: American Orthopaedic Foot and Ankle Society.

FAAM: Foot and Ankle Ability Measure.

#### 3.5.4 Upper limb

**Fig. 10:**
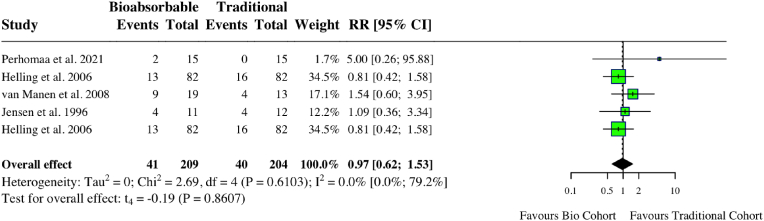
Forest plot (random effects) with accompanying risk ratio calculations and 95 % confidence intervals for complications comparing bioabsorbable and traditional implants in upper-limb studies.

**Fig. 11:**
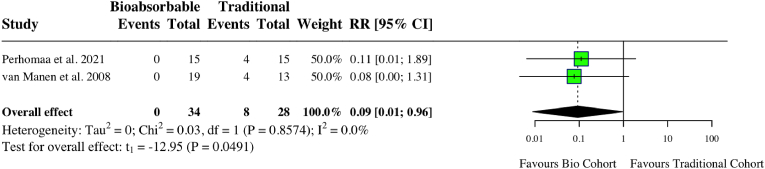
Forest plot (random effects) with accompanying risk ratio calculations and 95 % confidence intervals for presence of upper limb ongoing pain.

**Fig. 12:**
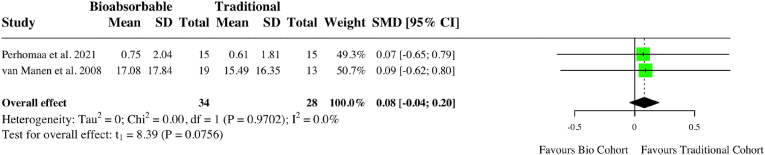
Forest plot (random effects) with accompanying mean difference calculations and 95 % confidence intervals for upper-limb DASH scores.

A meta-analysis of five studies assessing complications in upper limb surgery studies found no difference between groups (RR: 0.97, 95 % CI: 0.62, 1.53, *I*^*2*^ = 0 %, p = 0.86) (Fig. 10). A meta-analysis looking at two studies examining ongoing pain after upper limb fixation found less pain in the bioabsorbable group (RR: 0.09, 95 % CI: 0.01, 0.96, *I*^*2*^ = 0 %, p = 0.049) (Fig. 11). There was no difference in DASH score (MD: 0.08, 95 % CI: 0.04, 0.20, *I*^*2*^ = 0 %, p = 0.76) (Fig. 12). One study compared internal fixation with biodegradable versus smooth metal pins in pediatric lateral humeral condyle fractures and found a mildly reduced range of motion in 6 (54.5 %) of patients in the biodegradable group compared to 2 (25 %) in the metallic group, with neither being considered functionally significant.^29^ One patient in the biodegradable group experienced swelling (9.1 %), one in the metallic group had recurrent crepitus (12.5 %), and one in the metallic group had recurrent weakness (12.5 %). Individual study characteristics and outcomes can be seen in Table 4.

**Table 4:** Study characteristics and outcomes for upper-limb studies.

Author (Year)	Number of Patients Metal: M Bio: B	Mean follow-up time (years)	Lost to follow-up (%) Metal: M Bio: B	Female (%) Metal: M Bio: B	Mean age (years) Metal: M Bio: B	Implant type Metal: M Bio: B	Fixation	DASH score (SD) Metal: M Bio: B	Complication Metal: M Bio: B	Re-operation Metal: M Bio: B
Hope (1991)	M: 11 B: 13	0.5	M: 0 B: 0	M: 2 B: 5	M: 7.7 B: 8.5	M: Kirschner Wire B: PGA	Pins	NR	M: 9 B: 1	M: 5 B: 0
Perhomaa (2021)	M: 15 B: 15	6.8	M: 1 B: 4	M: 8 B: 10	M: 9.9 B: 10.2	M: Titanium B: PLGA	Screws	M: 0.75 (2.04) B: 0.61 (1.8)	M: 0 B: 2	NR
Helling (2006)	M: 82 B: 82	2	M: 21 B: 9	M: 28 B: 31	M: 38 B: 39	M: Metal B: PLA	Screws	NR	M: 16 B: 13	M: 3 B: 2
van Manen (2008)	M: 13 B: 19	1	NR	M: 10 B: 15	M: 60 B: 62	M: NR B: PLGA	Plate	M: 15.5 (16.4) B: 17.1 (17.8)	M: 4 B: 6	M: 4 B: 6
Jensen (1996)	M: 12 B: 11	0.5	M: 0 B: 0	M: 9	M: 47 B: 32	M: Stainless steel B: PDS	Pins	NR	M: 4 B: 4	M: 0 B: 1
				B: 9						

PLA: Polylactic acid.

PLGA: Poly lactic-co-glycolic acid.

PDS: Poly-p-dioxanone.

DASH: Disabilities of the Arm, Shoulder, and Hand.

NR: Not reported.

#### 3.5.5 Hip

One study examined screw fixation for iliac bone grafts for osteonecrosis of the femoral head and found a mean Harris score of 80.03 (5.21) for those who underwent bioabsorbable screw fixation, compared to 77.34 (5.15) for those with titanium fixation.^33^ Operation time in the bioabsorbable group was a mean of 175.4 min compared to 187.4 in the titanium group. No complications were found in either group at 6 months follow-up.

#### 3.5.6 Spine

One study comparing bioabsorbable and titanium plate fixation in cervical spine fusion found a mean VAS arm pain score at six months of 1.4 (1.4) and 1.2 (1.4) in each group, respectively.^34^ Neck Disability Index scores at six months were 0.11 (0.09) and 0.15 (0.07) in the bioabsorbable and titanium group, respectively. Bone density was 1090 and 1055 Hounsfield units in each group, respectively. There were no complications at six months in either group.

#### 3.5.7 Other

One study compared biodegradable and metallic screw plugs in olecranon and patella fractures.^35^ One patient who underwent biodegradable fixation in the patella group experienced a recurrent fracture, while two patients who underwent metallic fixation of the olecranon experienced dislocations.

## 4 Discussion

This systematic review and meta-analysis provides a comprehensive assessment of the safety and efficacy of bioabsorbable implants compared to traditional metal implants in orthopaedic surgery. The primary finding shows that bioabsorbable implants demonstrate comparable results across most anatomical locations in orthopaedic surgery and are a viable alternative to traditional metallic implants. There was a statistically significant reduction in surgical site infections in the bioabsorbable group, with no significant differences in overall complications, hardware failure, pain, functional outcomes, or time to union. A subgroup meta-analysis of those undergoing ankle surgery found that bioabsorbable implants were associated with a significant increase in complications. This was likely influenced by one study^21^ that found a FBR rate of 33.7 %. This study used uncut PLLA screws, which may have increased soft-tissue irritation and contributed to the higher rate of FBRs. Notably, 18 of the 29 FBRs reported were mild and resolved with local wound care. Excluding this outlier study in a sensitivity analysis eliminated the statistical significance. We also found comparable clinical outcomes and PROMs among both groups.

The advantages of biological implants in orthopaedic surgery are that they do not require removal and are radiolucent; hence do not obstruct radiographic imaging.^6^^,^^36^^,^^37^ A recent cost-effectiveness analysis in ACL reconstructions found positive cost-benefits of a proposed new bioabsorbable implant when it reduces the probability of failure by more than 30 %.^38^

In addition to highlighting the theoretical and practical advantages of bioabsorbable materials, it is essential to acknowledge the distinct complication profile, such as FBRs, osteolysis, and soft tissue damage associated with bioabsorbable materials.^39^ FBRs are one of the main complications of bioabsorbable implants, which involve an inflammatory response due to the acidic degradation of their byproducts. FBRs occur when the rate of degradation exceeds debris removal.^40^ These reactions can range from mild swelling and redness, which generally does not interfere with healing, to severe reactions requiring surgical debridement.^41^ Although sterile, FBRs can be associated with secondary bacterial infections, which often require antibiotics, hardware removal, irrigation and debridement, and potentially repeat fixation. The timeline of FBRs depends on the specific polymer used. A review found that PGA polymers can cause a clinical foreign-body reaction within 16 weeks of the operation; however, this did not interfere with healing.^42^ PLA polymers may cause FBRs up to five years later.^43^^,^^44^ Second-generation co-polymers and blends aim to provide further mechanical strength and prevent early adverse reactions; however, they still elicit FBRs. Third-generation implants incorporate biological materials such as magnesium, ceramic and hydroxyapatite, with the goals of minimizing adverse reactions and neutralizing acid degradation. Many studies included in this review included first and second-generation polymers, which were associated with higher FBR complications. However, the studies that included newer polymers showed significantly lower complication profiles, none of which included FBRs.^10^^,^^17^^,^^24^^,^^33^ A recent study incorporating hydroxyapatite into PLLA polymers in ACL reconstructions found no complications in the bioabsorbable group.^17^ Another study assessed implants for hallux valgus corrections and found no FBRs in the group who had magnesium implants.^24^ These studies highlight the promise of newer polymers, and future research should focus on assessing these implants.

Our findings align with previous research, which show comparable clinical outcomes when using bioabsorbable or metallic implants.^45^, ^46^, ^47^, ^48^, ^49^ The variance in complications and PROMs among different subgroups included in this review highlights the importance of considering the anatomical location when selecting implant materials, as bioabsorbable implants generally possess lower mechanical strength compared to metallic ones.^50^ In weight-bearing joints like the ankle, this difference is particularly significant, as the ankle endures significant mechanical loading which necessitates high implant integrity to withstand these stresses. One study reported that absorbable implants used in ankle fractures result in similar functional outcomes and complications compared with metal implants.^51^ However, due to the lower mechanical strength of bioabsorbable implants, strict adherence to post-operative instructions is crucial, as non-compliance could lead to implant failure or screw breakage.^52^ Bioabsorbable metals such as magnesium, iron and zinc exhibit higher mechanical strength than bioabsorbable polymers, making them more suitable for load-bearing applications; however, these metals have their own complications, such as hardening, PH disturbances, and unpredictable corrosion rates.^4^ Further research comparing bioabsorbable metals, bioabsorbable polymers, and traditional implants is warranted.

With respect to ACL reconstructions, previous studies suggest that bioabsorbable screws offer an increased risk of tunnel widening.^53^, ^54^, ^55^ In this review, overall complication and graft failure rates were similar between bioabsorbable and metallic implants. Furthermore, clinical outcomes were similar between both groups. One study with a 13-year mean follow-up reported no complications in the bioabsorbable group. However, a recent report highlighted two cases of subcutaneous pretibial pseudocyst formation after 7 and 10 years of successful anterior cruciate ligament reconstruction with PLLA bioabsorbable interference screws, underlining the presence of long-term complications.^56^ Due to the delayed absorption of certain materials, further long-term data is needed to elucidate the significance of these delayed reactions, along with how to mitigate them.

The strength of this systematic review is that it is a comprehensive analysis of all available RCTs comparing bioabsorbable to metallic implants in orthopaedic surgery. This review encompassed multiple subspecialties and associated outcomes while employing rigorous study methodology and utilizing multiple meta‐analyses. The limitations of this review are that some of the studies did not report specific details regarding the adverse events, which may have led to an underestimation of specific complications associated with the implants. Further, significant heterogeneity existed in some of the meta-analyses, which affected the confidence of the pooled findings. We also acknowledge that the surgeon's expertise and comfort with bioabsorbable implants may impact outcomes, and earlier studies with older polymers may have disproportionately affected the results. Lastly, there were differences in reporting of post-operative protocols between studies, which may have biased clinical outcomes and complication rates. There was only one comparative study on the hip and the spine, which diminishes the generalizability of the conclusions made in this paper with respect to these operations.

## 5 Conclusion

Bioabsorbable implants are a viable alternative to metallic implants in orthopaedic surgery, with similar complication rates and clinical outcomes. The success of these bioabsorbable implants is likely dependent on implant location, implant material, and surgeon expertise. Further long-term studies on newer generation biological polymers are needed to understand their optimal use in orthopaedic surgery.

## CRediT authorship contribution statement

**Benjamin Blackman:** Writing – original draft, Software, Methodology, Visualization, Investigation, Formal analysis. **Stefan Okunbor:** Writing – original draft, Visualization, Investigation, Formal analysis. **Aubrie M. Sowa:** Methodology, Writing – review & editing, Project administration. **Jake M. McDonnell:** Methodology, Conceptualization, Software, Writing – review & editing, Supervision, Project administration. **Tayler D. Ross:** Validation, Writing – review & editing. **Brian Rigney:** Validation, Writing – review & editing. **Stacey Darwish:** Conceptualization, Writing – review & editing, Supervision. **Joseph S. Butler:** Conceptualization, Writing – review & editing, Supervision.

## Informed consent

Not applicable.

## Ethical approval

This study does not contain any studies with human participants or animals performed by any of the authors.

## Funding

No funding was received for this study.

## Declaration of competing interest

The authors declare that they have no potential conflict of interest.
